# Tuberculosis active case finding: uptake and diagnostic yield among minibus drivers in urban South Africa

**DOI:** 10.1186/s12889-015-1592-x

**Published:** 2015-03-14

**Authors:** Tonderai Mabuto, Ephraim Zwane, Violet Chihota, Gillian Gresak, Salome Charalambous, Gavin J Churchyard, Christopher J Hoffmann

**Affiliations:** The Aurum Institute, Johannesburg, South Africa; School of Public Health, Faculty of Health Sciences, University of the Witwatersrand, Johannesburg, South Africa; London School of Hygiene and Tropical Medicine, London, UK; Division of Infectious Diseases, Johns Hopkins University School of Medicine, 725 N Wolfe St, Rm 226, Baltimore, Maryland 21205 USA

**Keywords:** Tuberculosis, HIV, Operational research, Transport, Transmission, Active case finding, Cross-sectional

## Abstract

**Background:**

Tuberculosis (TB) active case finding is a part of TB control in areas of higher TB prevalence. Congested public transportation settings may be areas of increased TB transmission. We evaluated the uptake and diagnostic yield of an active TB screening program among minibus drivers in a large public transportation facility in Johannesburg, South Africa.

**Methods:**

Over an eight month period, we intensively recruited minibus drivers for TB screening with a goal of 80% uptake among the estimated 2000 drivers. All participants were screened for TB symptoms, offered HIV testing, and had sputum collected for smear microscopy and liquid culture.

**Results:**

686 drivers were screened for TB, representing an uptake of only 34% of all drivers (43% of the target screening). Ten drivers (1.5%) were culture positive for TB, nine of whom were sputum smear microscopy negative. Factors associated with previously undiagnosed TB included a history of incarceration (odds ratio [OR] 5.5, 95% confidence interval: 1.1, 27.3) and HIV positivity (OR 5.3, 95% confidence interval: 1.1, 26.3).

**Conclusions:**

We identified undiagnosed pulmonary TB cases among drivers but at a level that may be insufficient to justify systematic case finding in this population considering the poor uptake.

## Background

Current approaches to tuberculosis (TB) control in low and middle income countries with high HIV and TB prevalence have achieved progress in reducing TB disease burden. However, prevalence and mortality rates are not declining sufficiently to achieve global targets set by the World Health Organisation (WHO) for 2015 [1]. This has been partly attributed to the challenge of early identification of infectious cases [2], especially in places or populations with poor access to health care, high levels of undetected TB, or a high risk of transmission [3,4]. These geographic or population-group clusters are sometimes referred to as TB ‘hot spots’ or transmission ‘catalysts’. They may include specific congregate communities or occupational risk environments that result in increased contact rates, exposure to highly infectious TB, or increased susceptibility to TB disease [5]. Identification of these settings, coupled with systematic targeted active case finding (ACF) and treatment efforts may lead to improved TB control [5].

In some situations, public transportation may serve as a catalyst for TB transmission. Public transportation is widely used in resource-limited-settings, especially among individuals living in communities that may have a higher prevalence of TB disease, including commuters from informal or low cost housing at the periphery of cities [6,7]. South Africa, which has among the highest prevalence of TB and HIV [2], and the fifth highest number of undiagnosed active TB cases [1], also has a high degree of dependence on public transportation. The majority of this transportation is provided by minibuses characterized by limited airspace, overcrowding, and variable ventilation [8]. Thus, minibuses may be places of increased TB transmission. In particular, minibus drivers may be at especially high risk of TB infection due to the confined airspace and exposure to many potentially infectious passengers [9-11].

The high risk of TB exposure and infection, a possibility of high levels of undetected TB, and long working hours that may limit access to health care may warrant implementation of ACF activities among minibus drivers. As part of service delivery within a transport center in central Johannesburg, South Africa, we provided walk-in HIV counselling and testing (HCT) coupled with TB screening to commuters and transport workers. Despite high overall utilization of these services, only 225 drivers (approximately 11% of the driver population) came for TB screening during the first four years of this service (unpublished Aurum data). The low uptake led to a reassessment of how best to reach drivers with TB screening. We decided to test a focused TB screening program prior to a scaled-up implementation. Specifically, we sought to provide a TB focused screening program for transport workers to measure uptake and the diagnostic yield of TB testing in a large transport center in central Johannesburg, South Africa.

## Methods

### Study setting

We implemented a TB ACF program based at a routine HCT and TB screening center in a transportation hub in central Johannesburg, South Africa from August 2011 to April 2012. The screening center was located in a space within the transport hub that was easily accessible to commuters and transport workers. The center served approximately 100,000 short-distance commuters daily who were traveling within greater Johannesburg and near-by towns or informal settlements [12]. During the study period, approximately 2,000 drivers operated daily from the transport center.

### The active case finding model

Prior to implementation of the ACF model, we obtained stakeholder buy-in from the transport center management, the 11 minibus associations operating within the center, and nearby public health clinics. In addition, we conducted focus group discussions with drivers to explore their perceptions regarding TB ACF and to identify ways to encourage TB screening. The formative interviews identified three key issues: (1) TB screening should be decoupled from HIV testing because drivers unwilling to undergo HCT were interested in TB screening, (2) TB screening needed to be brought to the driver, and (3) the total time required for the process needed to be short (<15 minutes).

We designed a program around these parameters. We provided screening in portable gazebos in minibus parking bays, limited the recipients of this service to transport workers to keep waiting queues short (routine HCT and TB screening was still available at the fixed center site), and made HIV testing optional and available at the end of the TB screening. The ACF team was comprised of two nurses who led a team of four trained lay counsellors. This team size allowed for screening 50 or more drivers during the main downtime in the drivers’ day, between 9 am and 3 pm, each in less than 15 minutes. Drivers were mobilized for screening through a combination of minibus association meetings, distribution of pamphlets, daily individual and group outreach, and provision of vouchers for food or fuel (valued at ZAR50/US$6).

TB screening was performed using the WHO four symptom tool (any duration of cough, fever, night sweats, or weight loss) [13]. In order to assess the sensitivity of the symptom screen in this population, a single spot sputum sample was collected from all drivers and sent to a single commercial laboratory for fluorescence microscopy and culture on mycobacterial growth indicator tubes (MGIT, Becton, Dickenson, & Company, New Jersey, USA).

Cultures that were positive for *Mycobacteria* species were identified as *M tuberculosis* or a non-tuberculosis mycobacteria species using Hain GenoType MTBDRplus and the Hain GenoType Mycobacterium CM (Hain Lifescience GmbH, Germany). Positive cultures were tested for drug susceptibility to first-line TB drugs using the Becton Dickinson BACTEC MGIT SIRE system (Becton-Dickinson, Franklin Lakes, USA). Participants were contacted telephonically to collect their TB microscopy results and when culture results were available. Individuals who were positive for TB were educated on the meaning of the test result and importance of treatment, referred to a health facility, and followed-up to ascertain entry into TB care.

All drivers were also offered HCT, but were not specifically required to undergo HCT to be screened for TB. HIV testing was done on-site using a rapid lateral flow assay (Determine® HIV-1/2, Alere, Massachusetts, USA) and, if positive, a confirmatory test was done using a different rapid lateral flow assay (Unigold™ Recombigen® HIV, Bray, Ireland). In addition, blood pressure screening and spot capillary blood glucose testing were offered to all drivers (Roche Accucheck® Active Test Kit, Basel, Switzerland).

Our goal was to screen 80% (1,600) of the minibus drivers at the transport center. We planned on a three month project period given the screening team capacity and goal. However, we extended screening an additional 5 months (total 8 months) due to low uptake.

### Study design

We performed a cross-sectional assessment. All participants were administered a brief questionnaire that included questions on demographics, working conditions, TB and HIV testing history, TB knowledge, attitudes and beliefs towards seeking health care, and current symptoms (any duration of cough, fever, night sweats, or weight loss). All participants completed a signed informed consent process prior to study procedures. The study was approved by the University of the Witwatersrand Human Subjects Ethics Committee.

### Analysis

We defined uptake by the total number of drivers screened as a proportion of the total population of drivers working at the transport center. Analyses of TB diagnosis were restricted to definite TB, defined as a sputum culture positive for *M. tuberculosis.* We excluded smear microscopy positive, culture negative TB from the analysis because of the limited specificity of a single sputum sample. We defined elevated random glucose as a random capillary blood glucose assay result of greater than 7.8 mmol/L [14]. We used logistic regression to assess for associations between undiagnosed prevalent TB and individual characteristics including sex, age, HIV status, duration of work, work hours, incarceration history, glucose level, and current smoking status. Incarceration history was defined as a participant reporting having spent at least one night in a police holding cell or in a correctional service facility. Multivariable logistic regression with backward elimination techniques was used for the final model. Retention of study variables was based upon a Chi-squared p-value of 0.05 or less.

## Results

### Participant characteristics and Uptake

From August 2011 to April 2012, 686 drivers were screened for TB (all of them were part of the evaluation). This represents an uptake of 34% (686/2000) of the drivers who were using this transport hub and 43% of the screening goal (686/1600). Among the 686 drivers, 684 (99.7%) were male with a median age of 37 years (interquartile range [IQR] 30–45; Table 1). The majority had worked at the transport center for more than a year and most (94%) worked more than five days a week for a median of 14 hours a day (IQR:13–15). They drove a median of six round trips per day (IQR: 5–8) with each leg of the trip averaging 30 to 60 minutes. At the time of screening, the majority, 424 (62%), reported ever having tested for HIV, while 406 (60%) agreed to HIV testing. Of those tested for HIV during the study, 59 of 406 (15%) tested positive (Table 1). A further 19 (2.8%) drivers who declined HCT reported a known HIV-positive status.

**Table 1:** **Characteristics of minibus drivers participating in an active TB screening program within in a large public transport facility in Johannesburg, South Africa**

	**N (%) or median (IQR)**
**Age, years**	37 (30, 45)
**Sex**	
Male	684 (99.7)
Female	2 (0.3)
**Detention**	
Ever detained	254 (37)
Never detained	432 (63)
**Current smoker**	
No	427 (62)
Yes	259 (38)
**Ever diagnosed with TB**	54 (8)
**TB treatment**	
Currently receiving treatment	4 (1)
Ever received treatment	50 (7)
Never on treatment	632 (92)
**Ever tested for HIV**	
Yes	424 (62)
No	262 (38)
Reported prior positive result	27 (6)
**HIV testing at encounter**	
HIV testing performed	406 (60)
Tested HIV positive	59 (15)
**Random glucose, mmol/L**	
<7.8 (normal)	547 (80)
7.8-11 (pre-diabetes)	93 (14)
>11 (diabetes)	40 (6)
**TB symptom screening**	
Cough	139 (20)
Fever	33 (5)
Night sweats	72 (11)
Weight loss	49 (7)
Cough, fever, night sweats, or weight loss	186 (27)

### TB screening results

We identified 10/686 (1.5%; 95% confidence interval [CI]: 0.7, 2.7) drivers with definite TB (Table 1), who were previously unaware of their TB-infection status. All specimens positive for TB were sensitive to isoniazid and rifampin. Only one of the culture positive TB cases was smear positive. A total of 186 (27%) drivers reported one or more of the four TB symptoms. The sensitivity and specificity of reporting one or more of the TB symptoms when compared to sputum culture as the gold standard was 80% (95% CI: 44, 96) and 74% (95% CI: 70, 77), respectively. Three additional drivers were not included among the TB cases; they were asymptomatic and had smear microscopy positive and culture negative results. An additional four drivers who were already diagnosed with TB and on TB treatment at the time of screening were not included among the TB cases.

### Factors associated with TB disease

In assessing risk factors for newly diagnosed TB disease we identified associations with the number of hours worked per day, having HIV infection, a history of incarceration, and currently smoking (Table 2). Elevated random blood glucose and a report of prior TB disease were not associated with a new TB diagnosis. In multivariable modelling, HIV infection (OR 5.3, 95% CI: 1.1, 26.3) and history of incarceration (OR 5.5, 95% CI: 1.1, 27.3) remained significantly associated with a new TB diagnosis.

**Table 2:** **Factors associated with TB disease among minibus drivers participating in an active TB screening program within in a large public transport facility in Johannesburg, South Africa**

	**†TB negative, N = 672 (%)**	**TB Disease, N = 10 (%)**	**Univariable odds ratio (95%** **confidence interval)**	**p**	**Multivariable odds ratio (95%** **confidence interval)**	**p**
**Age**						
<35	292 (43.4)	3 (30.0)	Referent	*0.9	Referent	*0.9
35-45	200 (29.8)	4 (40.0)	1.9 (0.4, 8.8)		1.7 (0.3, 8.3)	
>45	180 (26.8)	3 (30.0)	1.6 (0.3, 8.1)		1.7 (0.3, 9.2)	
**Hours worked per day**	14.2 (2.1)	15.4 (1.5)	1.5 (1.0, 2.2)	0.04	1.4 (0.9, 2.1)	0.07
**HIV status**						
Negative	343 (51.0)	3 (30.0)	Referent	1	Referent	1
Positive	55 (8.2)	4 (40.0)	8.3 (1,8, 38.2)	0.01	5.3 (1.1, 26.3)	0.03
Unknown	274 (40.8)	3 (30.0)	1.2 (0.3, 6.3)	0.8	1.0 (0.2, 5.2)	0.9
**TB history**						
No prior TB	624 (92.9)	8 (80.0)	Referent	1	-	-
Prior TB	48 (7.1)	2 (20.0)	3.4 (0.7,16.5)	0.12	-	-
**Incarceration history**						
Never detained	427 (63.5)	2 (20.0)	Referent	1	Referent	1
Ever detained	245 (36.5)	8 (80.0)	7.0 (1.5, 33.1)	0.02	5.5 (1.1, 27.3)	0.04
**Current smoker**						
No	421 (62.7)	3 (30.0)	Referent	1	Referent	1
Yes	251 (37.3)	7 (70.0)	3.9 (1.0, 15.3)	0.05	2.1 (0.5, 8.7)	0.3

*p value for trend.

†excludes four participants currently receiving treatment.

## Discussion

We found a substantial number of minibus drivers with pulmonary TB (1.5% with undiagnosed pulmonary TB). Importantly, we also encountered a major challenge in effectively engaging this population in ACF with an uptake of only 34% (versus a target of 80%) of minibus drivers.

The yield of TB diagnoses in our evaluation was approximately double that of a community-based mobile screening campaign performed in Western Cape, South Africa [15] but was less than that found in a larger community-based representative sample from the same region in South Africa in which 2.2% of adults were found to have culture positive pulmonary TB [16]. Importantly, our study was not a prevalence survey. We are unable to comment on the actual prevalence of TB among minibus drivers as we evaluated the feasibility of TB screening in the population and neither used random sampling nor achieved universal uptake.

We did not study factors associated with screening uptake. However, informal discussions with participants and non-participants during the course of the study revealed that poor uptake may have partly been attributable to the following: (1) fear of involuntary disclosure of TB results that could lead to loss of employment or discrimination within the workplace, (2) failure to see a benefit in TB diagnosis because of the unavailability of on-site TB treatment services, and (3) competing priorities for time during the off-peak period targeted for screening.

Prior studies have suggested an increased risk of TB disease among public transportation drivers and passengers. A study from Peru reported increased TB disease among commuters [11] and minibus drivers [10]. In addition, a mathematical model, based on assumptions from the South African context, predicted a 5% annual risk of TB for minibus passengers, assuming 400 annual trips per passenger [17]. It may be that the majority of the risk comes from other passengers because, in our study, the transport workers with TB were mostly sputum smear negative and less likely to transmit TB. Despite the possibility of a higher TB prevalence among this population, the combination of limited smear positivity and the extensive effort that only succeeded in screening a third of the drivers mean that screening activities aimed toward this population may not be an efficient use of resources.

The ten drivers with newly diagnosed TB worked slightly longer hours, were more likely to be HIV infected, and more likely to report a history of incarceration. HIV infection and incarceration in correctional facilities are both well documented to be associated with TB disease [18,19]. It is unclear whether the trend between TB and longer hours worked is due to increased contact with passengers with untreated pulmonary TB or whether it is confounded by other factors such as socio-economics. Caution should be used in interpretation of the multivariable analysis due to few TB cases.

This study has the advantage that it was a real-world implementation of TB ACF. As a result, we can describe challenges and total uptake of services. This framework introduces limitations on using the data for other purposes. Importantly, we are unable to accurately calculate the prevalence of TB in this population. It is unclear whether TB prevalence and risk profiles were similar among participants and non-participants. In addition, we only screened those who were currently working (screening occurred at the work place). Drivers missing work as a result of TB disease and other illnesses were not included (healthy worker effect). Furthermore, we lacked data on drivers who did not accept TB screening; thus we cannot compare characteristics between those who did and did not opt for screening.

## Conclusions

Ours is one of the few studies to assess TB screening among transport workers. While we identified substantial TB disease, it is unclear whether this differs from the background population from which the drivers came. Furthermore, willingness to be screened for TB was poor, compromising TB prevention efforts. Other approaches to hot spot or catalyst identification and screening may be more efficient for TB control than screening minibus drivers in South Africa.
